# Age, Height, and Sex on Motor Evoked Potentials: Translational Data From a Large Italian Cohort in a Clinical Environment

**DOI:** 10.3389/fnhum.2019.00185

**Published:** 2019-06-04

**Authors:** Mariagiovanna Cantone, Giuseppe Lanza, Luisa Vinciguerra, Valentina Puglisi, Riccardo Ricceri, Francesco Fisicaro, Carla Vagli, Rita Bella, Raffaele Ferri, Giovanni Pennisi, Vincenzo Di Lazzaro, Manuela Pennisi

**Affiliations:** ^1^Department of Neurology, Sant’Elia Hospital, ASP Caltanissetta, Caltanissetta, Italy; ^2^Department of Surgery and Medical-Surgical Specialties, University of Catania, Catania, Italy; ^3^Department of Neurology IC, Oasi Research Institute – IRCCS, Troina, Italy; ^4^Department of Neurology and Stroke Unit, IRCCS Centro Neurolesi Bonino Pulejo, Messina, Italy; ^5^Department of Internal Medicine, Sant’Anna Hospital, AUSL Reggio Emilia, Castelnovo ne’ Monti, Italy; ^6^Department of Medical and Surgical Sciences and Advanced Technologies, University of Catania, Catania, Italy; ^7^Research Unit of Neurology, Neurophysiology and Neurobiology, Università Campus Bio-Medico, Rome, Italy; ^8^Department of Biomedical and Biotechnological Sciences, University of Catania, Catania, Italy

**Keywords:** motor evoked potentials, transcranial magnetic stimulation, physical variables, reference values, central motor conduction time, translational neurophysiology

## Abstract

**Introduction:**

Motor evoked potentials (MEPs) to transcranial magnetic stimulation (TMS) are known to be susceptible to several sources of variability. However, conflicting evidences on individual characteristics in relatively small sample sizes have been reported. We investigated the effect of age, height, and sex on MEPs of the motor cortex and spinal roots in a large cohort.

**Methods:**

A total of 587 subjects clinically and neuroradiologically intact were included. MEPs were recorded during mild tonic contraction through a circular coil applied over the “hot spot” of the first dorsal interosseous and tibialis anterior muscles (TAs), bilaterally. Central motor conduction time (CMCT) was estimated as the difference between MEP cortical latency and the peripheral motor conduction time (PMCT) by cervical or lumbar magnetic stimulation. Peak-to-peak MEP amplitude to cortical stimulation and right-to-left difference of each parameter were also measured.

**Results:**

After Bonferroni correction, general linear (multiple) regression analysis showed that both MEP cortical latency and PMCT at four limbs positively correlated with age and height. At lower limbs, an independent effect of sex on the same measures was also observed (with females showing smaller values than males). CMCT correlated with both age (negatively) and height (positively) when analyzed by a single regression; however, with a multiple regression analysis this significance disappeared, due to the correction for the multicollinearity within the dataset.

**Conclusion:**

Physical individual features need to be considered for a more accurate and meaningful MEPs interpretation. Both in clinical practice and in research setting, patients and controls should be matched for age, height, and sex.

## Introduction

Transcranial magnetic stimulation is widely employed in daily clinical practice to non-invasively estimate *in vivo* and in real time the excitability of the M1 and the conductivity along the cortico-spinal tract. Moreover, the analysis of MEPs, produced contralaterally to the stimulated cortex, has recently attracting interest also in the assessment of synaptic plasticity and network connectivity, both in normal subjects and in patients with several neuropsychiatric disorders (Bella et al., 2011, 2013, 2016; Pennisi et al., 2015, 2016; Cantone et al., 2017; Lanza et al., 2017a), including systemic diseases involving the CNS (Pennisi et al., 2014; Bella et al., 2015). Briefly, TMS produces a rapid high-intensity pulse which passes unattenuated through the scalp (Hallett, 2007; Rossini and Rossi, 2007). When TMS is applied over M1, the cortex is activated through an electromagnetic induction, the impulses are transmitted along the cortico-spinal tract and peripheral nerves, so that a MEP can be recorded from a skeletal muscle using standard EMG surface electrodes. Translationally, MEPs provide a direct, objective, and painless assessment of the motor system (Hallett, 1996), including the excitability of the excitatory and inhibitory circuits, the integrity of central conduction pathways, and the functioning of transcallosal connections of motor cortices (Lanza et al., 2013).

Differentiating between altered MEP responses resulting from a central or peripheral nerve pathology and concomitantly excluding the sources of variability not related to neural dysfunction, is of paramount importance in clinical practice (Lanza et al., 2017b). Therefore, the reliable identification of normal or abnormal MEPs requires a comprehensive characterization in appropriate populations. Based on previous studies, some physical variables (i.e., age, height, and sex) all showed to affect MEPs (Chu, 1989; Booth et al., 1991; Ghezzi et al., 1991; Furby et al., 1992; van der Kamp et al., 1996; Mills and Nithi, 1997), although the samples studied are relatively small and conflicting evidences on the relationship between MEPs and individual characteristics have been reported. Additionally, most studies concentrated on the 20–50 years age range, and no conclusive description of reference values of upper and lower limb over different ranges of age (especially in older adults (Matamala et al., 2013) in a substantial sample of male and female subjects is available. Finally, several technical and procedural factors (such as the characteristics of the stimulator, the coil design, and other experimental conditions) make it difficult to obtain normative data and to compare those established by different laboratories.

To date, the relationship between MEPs and source of variability is not fully understood. If, in the same laboratory and under the same experimental conditions, a relationship between physical variables and MEPs is found, then, accounting for these factors through proper scaling of MEP parameters would allow for a more accurate recording and meaningful interpretation. Till now, however, no previously published study has systematically addressed these variables at the same time together. Correlating MEP cortical latencies with CMCT and height was suggested as an approach for standardizing MEPs response (Booth et al., 1991), although normal values and age- or height-adjusted latencies were not reported. About the influence that gender might have on conduction velocity, MEPs cortical latencies were found to be longer in males than in females (Mills and Nithi, 1997), albeit the possible confounding effect of height (in terms of longer conduction pathway in males) was not adequately addressed. Therefore, a systematic investigation of the effect of height on MEP cortical latency and CMCT between sexes and across different age groups is also warranted.

In the present study, diagnostic TMS data from a large cohort of subjects clinically and neuroradiologically intact are provided. Then, we assessed the relationship between MEPs and some physical variables (age, height, and sex) in order to identify the factors that are likely to affect motor responses. Given the physiological age-related slowing of the conduction velocity and the different length-dependent velocities between upper and lower limb, we hypothesized that both age and height would positively correlate with MEP cortical latency and PMCT. For the same reasons, we also expected a negative correlation between MEPs amplitudes and age, especially for lower limbs. When subjects’ height is considered, we hypothesized that the adjusted latencies should demonstrate minimal interindividual variability.

## Materials and Methods

### Participants

A total of 587 consecutive subjects ranging from 18 to 87 years in age (41.1% males) and from 145 to 197 cm in height were retrospectively included from the TMS Lab of the University of Catania (Italy), from March 2008 to November 2018. According to the inclusion criteria, none of them had motor deficit or history of central and peripheral motor or neuromuscular disorder based on a preliminary interview, a specific medical questionnaire, and a full neurological examination. All subjects had normal mobility and were able to engage in tasks of daily life without assistance, even the most elderly. Any CNS pathology was also ruled out by brain and spinal magnetic resonance imaging. Therefore, all participants eventually included were neurologically intact.

Based on previous TMS studies (Livingston et al., 2010, 2013; Matamala et al., 2013; Cueva et al., 2016), subjects were excluded if they had: history or presence of epilepsy, moderate-to-severe traumatic head injury, previous cranial or spinal surgery, stroke or chronic cerebrovascular diseases, chronic pain syndrome, peripheral neuropathies or other neurological or neuromuscular disorders; current or previous psychiatric diseases; any acute, advanced, or chronic not compensated medical illness (including diabetes, hypothyroidism, and neoplasm); alcohol or drug abuse; implanted electrical biomedical devices (i.e., pacemaker), pregnancy at the time of testing, or any other contraindication to TMS (Rossi et al., 2009); current treatment with neuroactive drugs or any other medication able to affect cortical excitability (Paulus et al., 2008; Ziemann et al., 2015). Out of 587, 482 were out-patients, mainly referred by general practitioners or other specialists for non-specific clinical complaints in order to rule out the possibility of an underlying neurological condition. The remaining 105 were in-patients admitted because of subjective motor symptoms without clinical, radiological, and neurophysiological correlates.

Height was measured with a cloth tape measure with the subject standing in the anatomical position (barefoot, with heels together, arms at the side, legs straight, shoulders relaxed, and head in the horizontal plane). Measurement was recorded to the nearest 0.1 cm.

This study was carried out in accordance with the recommendations of the guidelines of the International Federation of Clinical Neurophysiology Committee for the diagnostic use of TMS (Ziemann et al., 2015). The protocol was approved by the Ethics Committee of the “Azienda Ospedaliero Universitaria Policlinico-Vittorio Emanuele” of Catania, Italy. All subjects gave written informed consent in accordance with the Declaration of Helsinki of 1964 and its later amendments.

### Instrumentations and Technical Considerations

A high-power monopulse biphasic electromagnetic stimulator MagStim 220 (The Magstim Co., Ltd., Whitland, Dyfed, United Kingdom) capable of generating a maximal output of 2.0 Tesla, with a maximum duration of <1 ms and a rise time of 100 μs, was used to evoke motor responses. Magnetic pulse intensity was expressed as a percentage of the maximal stimulator output (100%). The capacitor was connected to a 90 mm circular coil (inner diameter of 5 cm), routinely employed for diagnostic TMS. Since the round coil stimulates a larger cortical volume, the positioning over the target region is easier than with the focal “figure-of-eight” shaped coil. The large round coil also results in a better depth penetration, which is advantageous for TMS of M1 leg area. Finally, the round coil is less susceptible to the unavoidable minimal changes in the coil position (Groppa et al., 2012; Rossini et al., 2015).

Coil was applied with the handle pointing backward and held tangentially flat on the scalp, with its center positioned over Cz (according to the international EEG 10–20 system) for recording from the FDI and over Fz for recording from the TA. For TMS of the right hemisphere, the current direction within the circular coil was clockwise, so that the induced cortical current was perpendicular to the cortex in posterior-anterior direction, and vice versa for the left hemisphere, as recommended (Wassermann et al., 2008). After the location was identified, the coil position was slightly adapted until the best excitation point (“hot spot”) was accomplished. Once the position was defined, the outer rim of the coil was marked with a dermographic pen on the scalp to enable the examiner to maintain a constant position.

All motor responses were obtained at 80% of the maximum stimulator output, based on the evidence that threshold stimulation for a 2.0 Tesla magnetic stimulator is about 50–65% of the maximal output (Amassian et al., 1989; Alexeeva et al., 1998; Garry et al., 2004). In such a way, a visible contraction of the target muscle was constantly observed after each stimulation. We also verified that MEP cortical latency did not further shorten and amplitude did not further increase by incrementing the intensity above 80%. This implies that the intensity used was sufficiently high to excite the fast-conducting cortico-spinal neurons (Groppa et al., 2012).

Motor responses were amplified and filtered (bandwidth 3-3,000 Hz) using a 2-channel Medelec Synergy system (Oxford Instruments Medical, Inc., United Kingdom), with an amplification factor of the screen of 1 mV/division unit during the MEP recording. The temporal resolution of the screen (sweep) was 5 ms/division unit, in such a way that the TMS artifact, the beginning and the end of MEP were always clearly visible.

### Subject Preparation

A detailed explanation of the exam was preliminarily provided to each subject. In preparation for placement of the recording electrodes and to decrease cutaneous impedances, the skin was gently abraded with fine-grade sandpaper and cleaned with an isopropyl alcohol pad. MEPs were recorded via standard surface EMG silver/silver chloride cup electrodes (9 mm diameter), filled with electrode jelly and applied on FDI and TA contralaterally to the side of stimulation, in a conventional belly tendon montage. For upper limbs, the recording (active) electrode was placed over the mid-point of the FDI belly, the reference electrode distally at the metacarpal-phalangeal joint of the index finger, and the ground electrode on the radial surface of wrist; for lower limbs, the recording (active) electrode was placed over the mid-point of TA belly, the reference electrode 3–4 cm distally over the muscle tendon, and the ground electrode over the patella. The FDI muscle, commonly examined using TMS, was selected because it can be easily contracted and recorded compared to other hand muscles. Based on the fact that evoking MEPs in the lower limbs is usually more difficult than in the upper limbs, we used the TA muscle for a number of reasons: it has a more pronounced representation than most of the other leg muscles; it has a relatively low excitation threshold; its MEPs have a larger amplitude compared to other leg muscles (Petersen et al., 2003); differently from the foot muscles, it is usually not wasted in elderly patients (Claus, 1990). Electrode impedance was constantly kept <10 KOhms, as recommended (Groppa et al., 2012).

Side-to-side difference was also considered, with “right” and “left” referred to the recording side of the target muscle. Trials containing any type of artifact were removed. Similarly, we have excluded trials contaminated by EMG activity at rest (indicating a non-relaxed muscle), as well as the “active” trials (during contraction) with excessive EMG voluntary activity that made a reliable recognition of the onset of MEP cortical latency difficult or doubtful.

All data were collected on a dedicated PC and stored for off-line analysis. Subjects were seated in a comfortable armchair, in a quiet environment, and asked to keep their hands and legs as relaxed as possible. All exams were conducted in the same laboratory and experimental conditions (including room temperature), at the same time of the day (approximately 9:00–11:30 am) and by the same trained operators. All measurements were made by a senior operator (GL) and finally checked and approved by the Lab head (GP).

### TMS and Spinal Magnetic Stimulation

First, a reference MEP to TMS in the relaxed muscle was obtained. Then, subjects were asked to produce a small transient tonic contraction of the target muscle (about 10–20% of the subject’s maximum voluntary contraction, just enough to overcome gravity), in order to obtain MEPs with higher amplitude and shorter latency compared to the reference response. Contracted MEPs, indeed, are mediated by the large and fast-propagating α-motoneuron pools and reflect a fast-propagating system from the cortex to the muscle (Rossini et al., 2015). Since active contraction potentiates and stabilizes MEPs (Boroojerdi et al., 1999; Sohn and Hallett, 2004), five trials were sufficient to confirm their reproducibility (Rossini and Caramia, 1992). Muscle contraction was kept constant by using a strain gauge and with the help of a continuous auditory and visual EMG activity monitoring, as recommended (Fritz et al., 1997). The acoustic feedback also allowed to monitor the level of muscular activity and to check for complete relaxation (Rossini et al., 2015).

Motor evoked potential cortical latency was calculated as the time interval from the TMS artifact to the first negative deflection of the muscular response from EMG baseline (Rossini et al., 2015). The MEP with the shortest latency was considered for CMCT calculation, according to international guidelines. Similarly, since diagnostic TMS estimates the cortico-motor response with maximal amplitude, only the trial with the largest amplitude was used for MEP size analysis. Amplitude was measured from the maximal negative to maximal positive deflection of the selected MEP (peak-to-peak amplitude) (Groppa et al., 2012). MEP amplitude represents the final pathway of spatial and temporal summation of several descending volleys activating the α-motoneurons, thus reliably reflecting the excitation state of the cortico-spinal cells, the pyramidal tract, the peripheral motor nerve, and the target muscle (Rossini et al., 2015).

Peripheral stimulation of the motor roots was carried out in all subjects to determine PMCT. MEPs to cervical or lumbar stimulation are presumably elicited by a direct ventral root excitation (Mills and Murray, 1986) and have been shown to display similar latencies when either magnetic or electric stimuli are applied (Caramia et al., 1989). In order to stimulate magnetically the spinal roots and facilitate foraminal stimulation, subjects were requested to slightly bend the neck or the trunk forward. The center of the coil was placed posteriorly over the 7th cervical (for upper limbs) and 4th lumbar (for lower limbs) spinous process. In some cases, the coil was slightly shifted laterally to the same side of the target muscle to define the location where maximum responses could be obtained, or slightly moved vertically up and down to determine the most effective level for stimulation. In any case, coil location and orientation were such that the maximal induced current flowed horizontally in the tissue toward the midline from the ipsilateral side of the muscle (Mills et al., 1993). Unlike stimulation of M1, facilitation is not needed for spinal stimulation (Claus, 1990), and, therefore, subjects were recorded at rest. PMCT was calculated as the time interval from the TMS artifact to the first negative spike from EMG baseline. To ensure reliability, two reproducible responses were recorded and averaged (Rossini et al., 2015).

Central motor conduction time was defined as the conduction time from motor cortical neurons to spinal motor neurons, thus reflecting the conductivity along the cortico-spinal tract (from the upper to the lower motor neuron). CMCT was estimated by subtracting the peripheral (cervical or lumbar) PMCT from the shortest MEP cortical latency (Rossini et al., 1985a,b, 1987, 1994; Ugawa et al., 1994): CMCT = MEP cortical latency – PMCT. CMCT is measured with the target muscle active, thereby giving the shortest latency from the cortex to the muscle. In this situation, the spinal motoneuron pool is close to the firing threshold and there is the greatest opportunity for the earliest descending cortico-spinal volley to induce a discharge (Chen et al., 2008).

### Statistical Analysis

We first assessed the normality of the distribution of each variable under consideration in the whole group of subjects by mean of the Kolmogorov–Smirnov and the Lilliefors tests for normality. We then checked for possible simultaneous effects of age, height, and sex (independent factors) on the variables under consideration (dependent variables) by means of the General Regression Models module offered by the commercially available software STATISTICA v.6 (2001), StatSoft Inc., (this software was also used for all other statistical tests carried out in this study). For each study variable, three partial correlation coefficients were obtained, one for each independent factor, together with its statistical significance. Because of the large number of partial correlation coefficients obtained, we only considered as being significant the *p*-values that continued to be <0.05 after the Bonferroni correction (Bland and Altman, 1995). Due to the high number of subjects included, also small correlation values tend to be significant; however, following the Cohen’s (Cohen, 1988) indications, we considered correlations 0.10, 0.30, and 0.50 as corresponding to small, medium, and large sizes, respectively, and considered only correlations ≥0.30 for further analysis. After this step, we computed descriptive statistics for all variables in the whole group (mean, standard deviation, mean ± 1.96 SD, and 95% confidence interval). For variables showing a moderate-to-large partial correlation coefficient with age and/or height and/or sex, subgroup specific scatterplots were obtained.

## Results

### Descriptive Results

**FIGURE 1:**
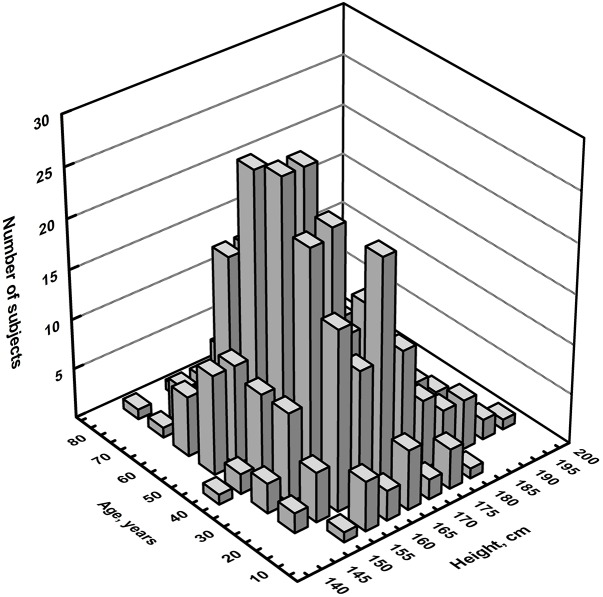
Three-dimensional histogram of the distribution of the number of subjects per age and height.

Both TMS and spinal root stimulations were well tolerated and no side-effect or significant discomfort was reported during or after the exam. As shown in Figure 1, no skewed distribution of age and height was present in the sample. Similarly, in the whole sample of subjects, the difference in height between males and females was not statistically significant, whereas, as expected, there was a decline in the mean height in both sexes as age increased. In all subjects, motor responses during active contraction of FDI and TA were always obtained and recorded. Although MEPs from the lower limbs were usually more difficult to elicit than those recorded from the hand (Barker et al., 1987), we did not experience significant difficulty.

Table 1 summarizes the demographic features of all participants and the age subgroups. Mean value ± SD and the 95% confidence intervals for each measure, in the whole sample and split by muscle and side, are summarized in Table 2. The upper and lower limits were derived by determining the cut-off scores of 1.96 SD above and below the mean value, although these represented unadjusted latencies (not correlated for age, height, and sex). Table 3 shows descriptive statistics of MEP cortical latency and PMCT computed separately for each age group.

**Table 1:** Demographic features of the whole group of participants and of the age subgroups.

		n	Mean	SD
**Age ≥ 18 < 35 years**	F	91	26.8	4.83
	M	63	25.8	4.86
**Age ≥ 35 < 50 years**	F	138	41.7	4.05
	M	68	42.2	4.02
**Age ≥ 50 < 65 years**	F	83	55.8	3.64
	M	71	57.1	4.35
**Age ≥ 65 years**	F	34	70.6	6.32
	M	39	69.7	4.12
**All**	F	346	44.0	14.23
	M	241	46.7	16.05
**Total**		587	45.1	15.05


*n, number of subjects; SD, standard deviation; F, female; M, male.*

**Table 2:** Descriptive statistics of all variables studied.

	Mean ± SD	Mean ± 1.96 SD	95% confidence interval
**Right FDI**			
*MEP amplitude, mV*	7.9 ± 3.21	1.6/14.2	2.8/15.1
*MEP cortical latency, ms^∗^*	19.5 ± 1.45	16.7/22.4	16.9/22.5
*PMCT, ms^∗^*	13.6 ± 1.31	11.0/16.2	11.2/16.2
*CMCT, ms^∗^*	5.9 ± 0.89	4.2/7.7	4.3/7.6
**Left FDI**			
* MEP amplitude, mV*	7.6 ± 3.09	1.6/13.7	3.0/14.6
*MEP cortical latency, ms^∗^*	19.4 ± 1.45	16.6/22.2	17.0/22.5
*PMCT, ms^∗^*	13.5 ± 1.32	10.9/16.1	11.2/16.0
*CMCT, ms^∗^*	5.9 ± 0.87	4.2/7.6	4.2/7.6
**Right-Left difference**			
*MEP amplitude, mV*	0.26 ± 2.23	-4.1/4.6	-4.0/4.9
*MEP cortical latency, ms*	0.12 ± 0.69	-1.2/1.5	-1.3/1.5
*PMCT, ms*	0.09 ± 0.62	-1.1/1.3	-1.3/1.3
*CMCT, ms*	0.026 ± 0.75	-1.4/1.5	-1.5/1.5
**Right TA**			
*MEP amplitude, mV*	5.5 ± 2.30	1.0/10.0	2.0/10.4
*MEP cortical latency, ms^∗^*	26.5 ± 2.21	22.2/30.9	22.7/31.2
*PMCT, ms^∗^*	12.7 ± 1.43	9.8/15.5	10.2/15.8
*CMCT, ms^∗^*	13.9 ± 1.64	10.7/17.1	10.9/17.1
**Left TA**			
* Amplitude, mV*	5.3 ± 2.17	1.1/9.6	1.9/10.0
*MEP cortical latency, ms^∗^*	26.5 ± 2.20	22.2/30.8	22.7/31.0
*PMCT, ms^∗^*	12.6 ± 1.46	9.7/15.4	10.1/15.9
*CMCT, ms^∗^*	13.9 ± 1.69	10.6/17.2	10.8/17.2
**Right-Left difference**			
*MEP amplitude, mV*	0.18 ± 2.02	-3.8/4.1	-4.0/4.5
*MEP cortical latency, ms*	0.07 ± 1.94	-3.7/3.9	-4.4/4.1
*PMCT, ms*	0.07 ± 1.23	-2.3/2.5	-2.7/2.7
*CMCT, ms*	0.0002 ± 1.59	-3.1/3.1	-3.5/3.0


*^∗^Subgroup-specific graphs in Figure 2–10; SD, standard deviation; FDI, first dorsal interosseous muscle; TA, tibialis anterior muscle; MEP, motor evoked potential; PMCT, peripheral motor conduction time; CMCT, central motor conduction time.*

**Table 3:** Descriptive statistics of the MEP cortical latency and PMCT values computed separately for each age group.

	Mean	SD	Mean ± 1.96 SD	95% confidence interval
**Age ≥ 18 < 35 years**				
*Right FDI MEP cortical latency, ms*	19.2	1.43	16.4/22.0	16.5/21.9
*Right FDI PMCT, ms*	13.1	1.26	10.6/15.6	10.6/15.9
*Left FDI MEP cortical latency, ms*	19.1	1.41	16.3/21.8	16.4/22.0
*Left FDI PMCT, ms*	13.1	1.25	10.6/15.5	10.8/15.8
*Right TA MEP cortical latency, ms*	26.0	2.41	21.3/30.7	22.4/31.4
*Right TA PMCT, ms*	12.2	1.38	9.5/14.9	9.9/15.4
*Left TA MEP cortical latency, ms*	25.9	2.33	21.4/30.5	22.0/30.1
*Left TA PMCT, ms*	12.1	1.27	9.6/14.6	10.0/14.9
**Age ≥ 35 < 50 years**				
*Right FDI MEP cortical latency, ms*	19.5	1.40	16.8/22.2	17.1/22.5
*Right FDI PMCT, ms*	13.6	1.21	11.2/16.0	11.7/16.2
*Left FDI MEP cortical latency, ms*	19.3	1.38	16.6/22.0	17.1/22.4
*Left FDI PMCT, ms*	13.4	1.27	10.9/15.9	11.3/15.9
*Right TA MEP cortical latency, ms*	26.4	1.95	22.5/30.2	22.9/30.5
*Right TA PMCT, ms*	12.6	1.38	9.9/15.3	10.4/15.6
*Left TA MEP cortical latency, ms*	26.4	2.07	22.4/30.5	23.0/31.0
*Left TA PMCT, ms*	12.7	1.47	9.8/15.6	10.3/15.8
**Age ≥ 50 < 65 years**				
*Right FDI MEP cortical latency, ms*	19.7	1.39	17.0/22.4	17.4/22.8
*Right FDI PMCT, ms*	13.9	1.27	11.4/16.4	11.5/16.2
*Left FDI MEP cortical latency, ms*	19.6	1.42	16.8/22.4	17.2/22.7
*Left FDI PMCT, ms*	13.8	1.23	11.4/16.2	11.5/16.0
*Right TA MEP cortical latency, ms*	27.0	2.26	22.5/31.4	23.0/31.5
*Right TA PMCT, ms*	12.9	1.46	10.0/15.8	10.2/16.0
*Left TA MEP cortical latency, ms*	26.7	2.15	22.5/30.9	23.1/31.2
*Left TA PMCT, ms*	12.8	1.57	9.7/15.8	10.1/16.3
**Age ≥ 65 years**				
*Right FDI MEP cortical latency, ms*	20.0	1.59	16.9/23.1	16.9/23.1
*Right FDI PMCT, ms*	14.2	1.35	11.5/16.8	11.9/17.0
*Left FDI MEP cortical latency, ms*	19.9	1.56	16.9/23.0	17.0/23.3
*Left FDI PMCT, ms*	14.2	1.37	11.5/16.8	11.4/16.9
*Right TA MEP cortical latency, ms*	27.3	2.06	23.2/31.3	22.6/31.3
*Right TA PMCT, ms*	13.1	1.34	10.5/15.7	10.3/16.3
*Left TA MEP cortical latency, ms*	27.2	2.13	23.1/31.4	22.7/31.6
*Left TA PMCT, ms*	12.9	1.30	10.4/15.5	10.1/15.3


*Subgroup-specific graphs for all these parameters in Figure 2–10; S.D, standard deviation; FDI, first dorsal interosseous muscle; TA, tibialis anterior muscle; MEP, motor evoked potential; PMCT, peripheral motor conduction time.*

### Correlation Results

**FIGURE 2:**
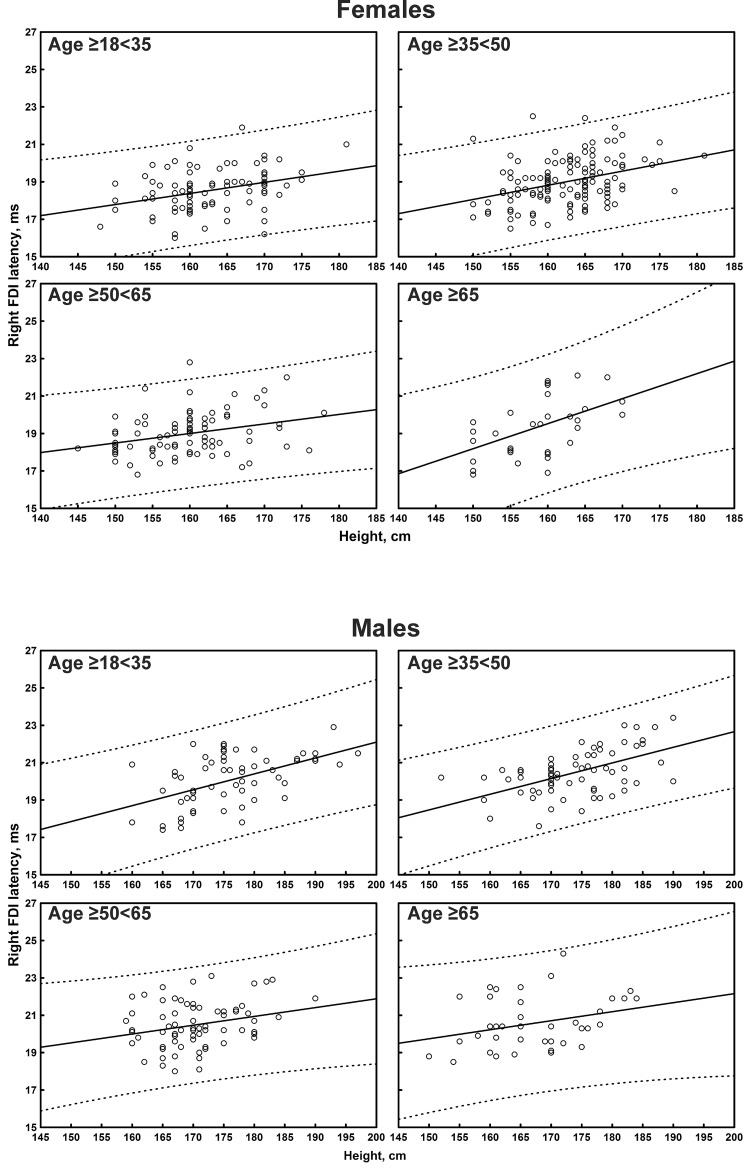
Correlation between age and right first dorsal interosseous muscle (FDI) cortical latency of the motor evoked potentials (MEPs) in each age subgroup of participants, divided by sex. The continuous line is the regression line while the two dashed lines represent the limits of the area within which the 95% of points are expected.

**FIGURE 3:**
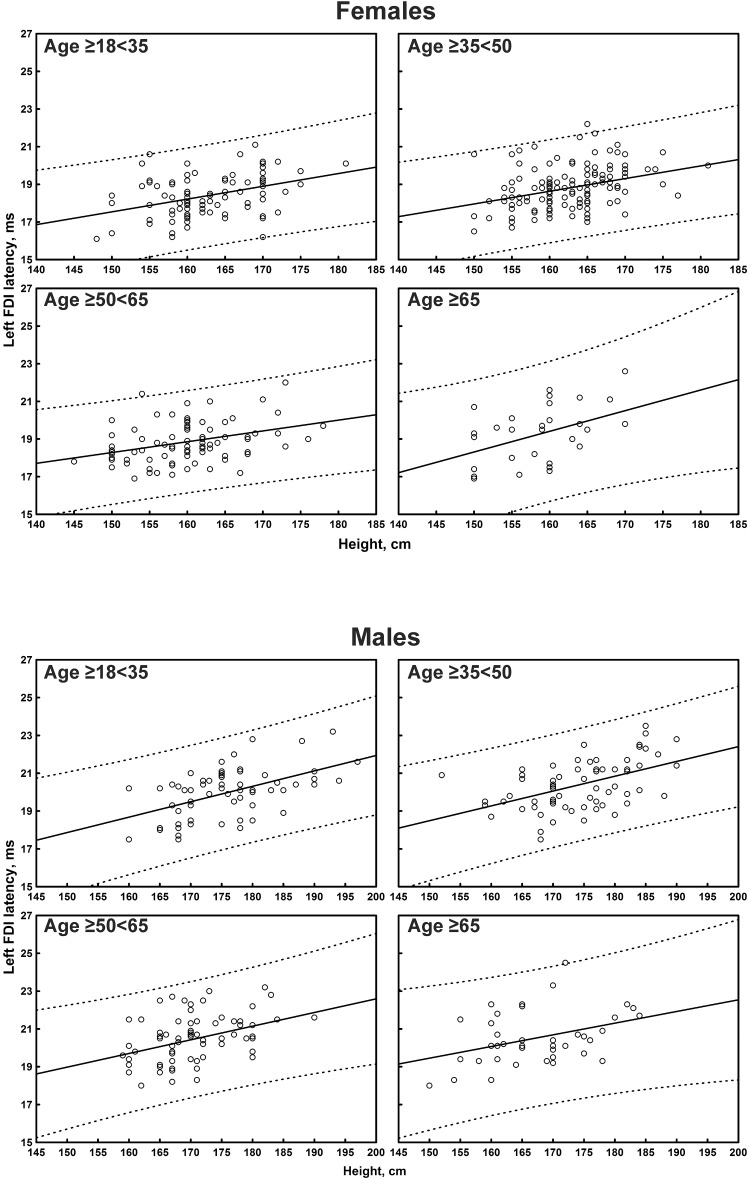
Correlation between age and left first dorsal interosseous muscle (FDI) cortical latency of the motor evoked potentials (MEPs) in each age subgroup of participants, divided by sex. The continuous line is the regression line while the two dashed lines represent the limits of the area within which the 95% of points are expected.

**FIGURE 4:**
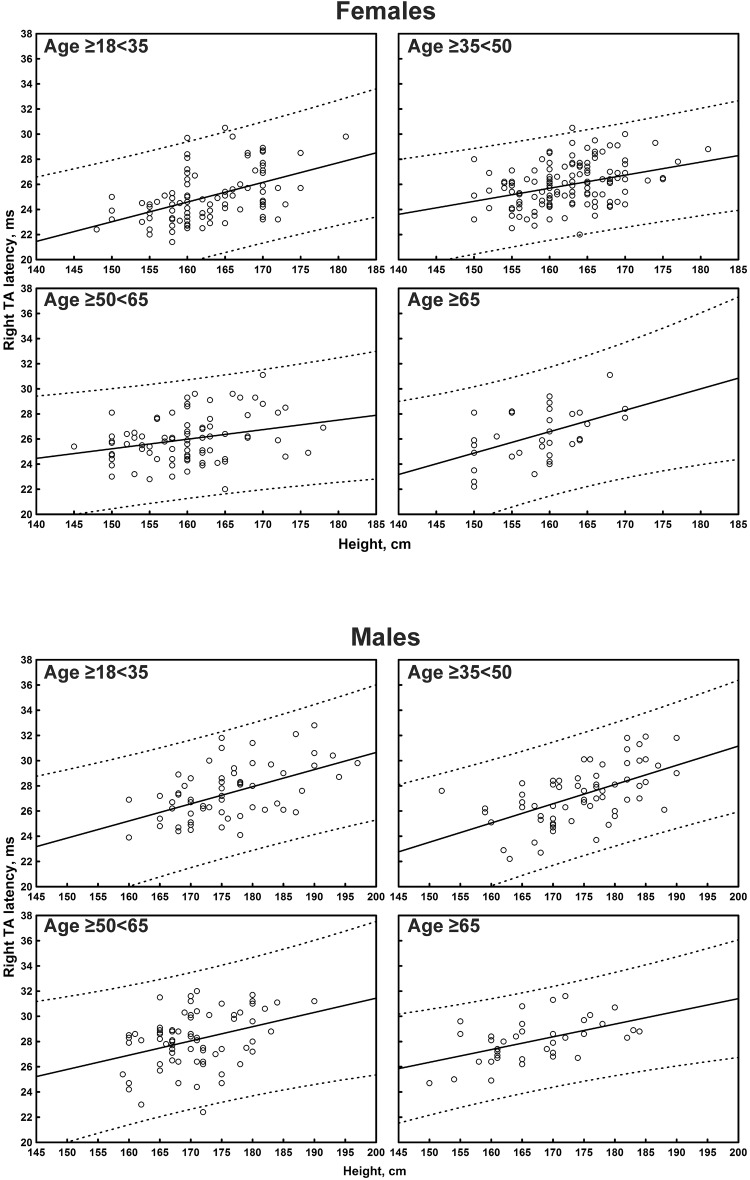
Correlation between age and right tibialis anterior muscle (TA) cortical latency of the motor evoked potentials (MEPs) in each age subgroup of participants, divided by sex. The continuous line is the regression line while the two dashed lines represent the limits of the area within which the 95% of points are expected.

**FIGURE 5:**
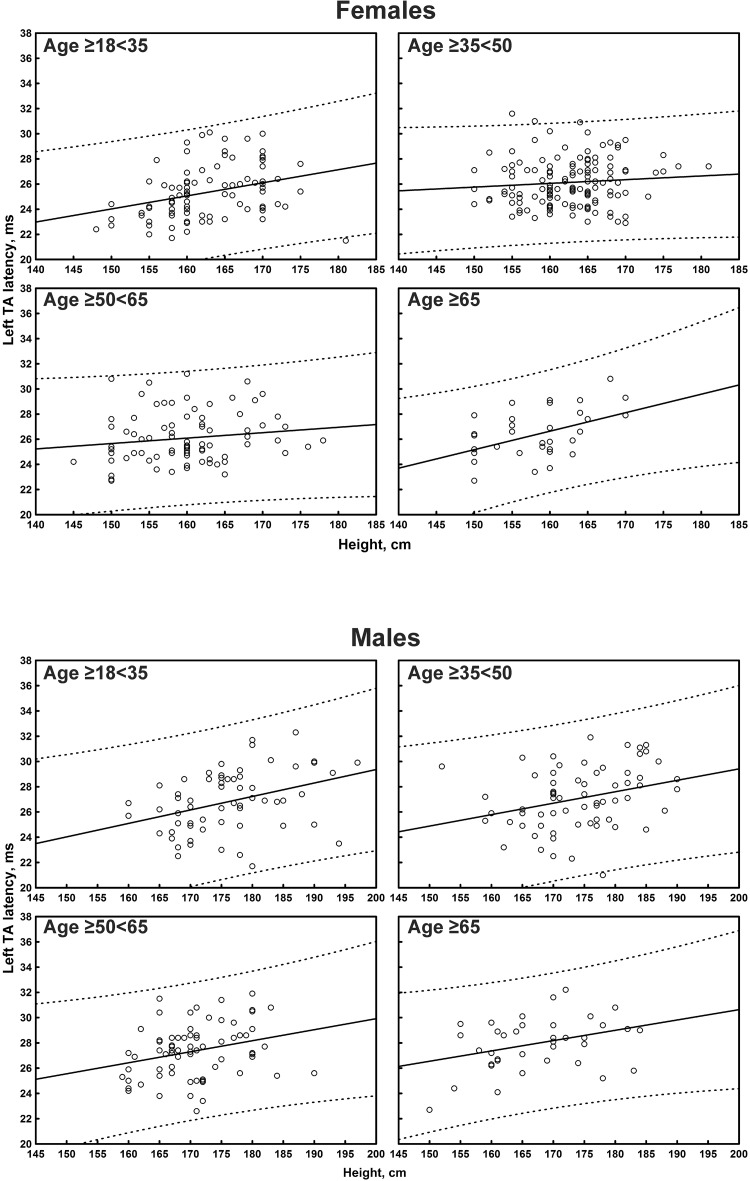
Correlation between age and left tibialis anterior muscle (TA) cortical latency of the motor evoked potentials (MEPs) in each age subgroup of participants, divided by sex. The continuous line is the regression line while the two dashed lines represent the limits of the area within which the 95% of points are expected.

**FIGURE 6:**
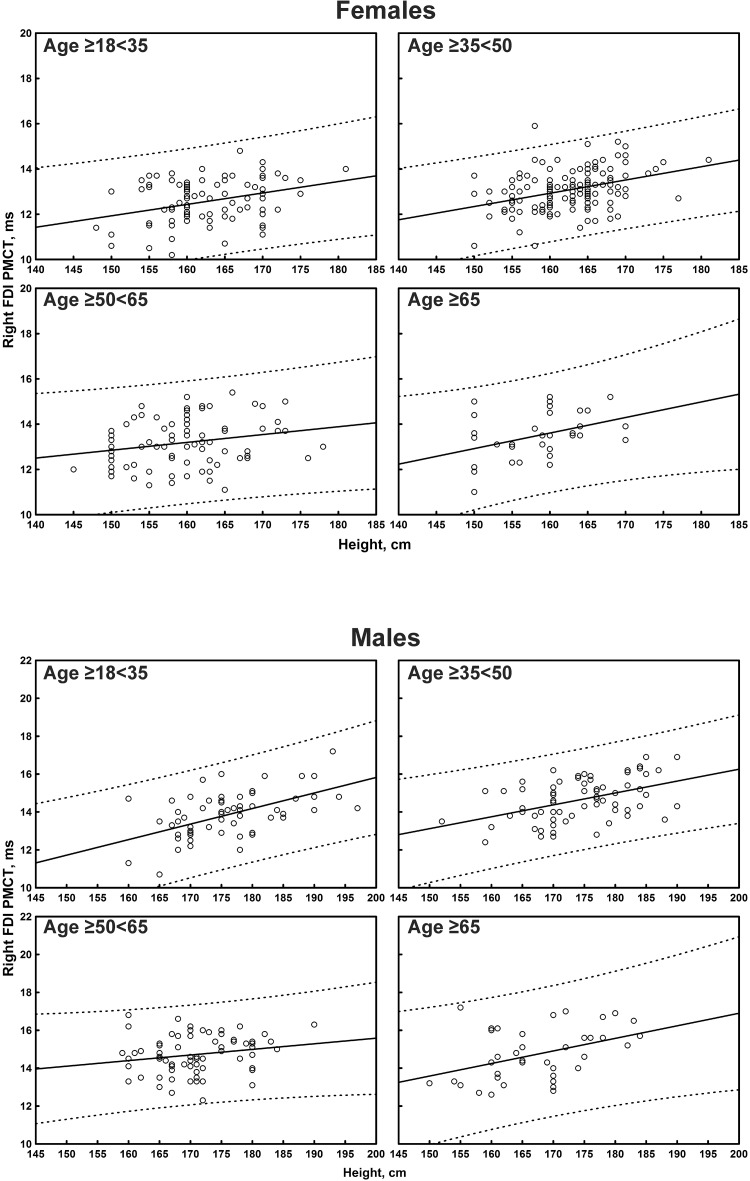
Correlation between age and right first dorsal interosseous muscle (FDI) peripheral motor conduction time (PMCT) in each age subgroup of participants, divided by sex. The continuous line is the regression line while the two dashed lines represent the limits of the area within which the 95% of points are expected.

**FIGURE 7:**
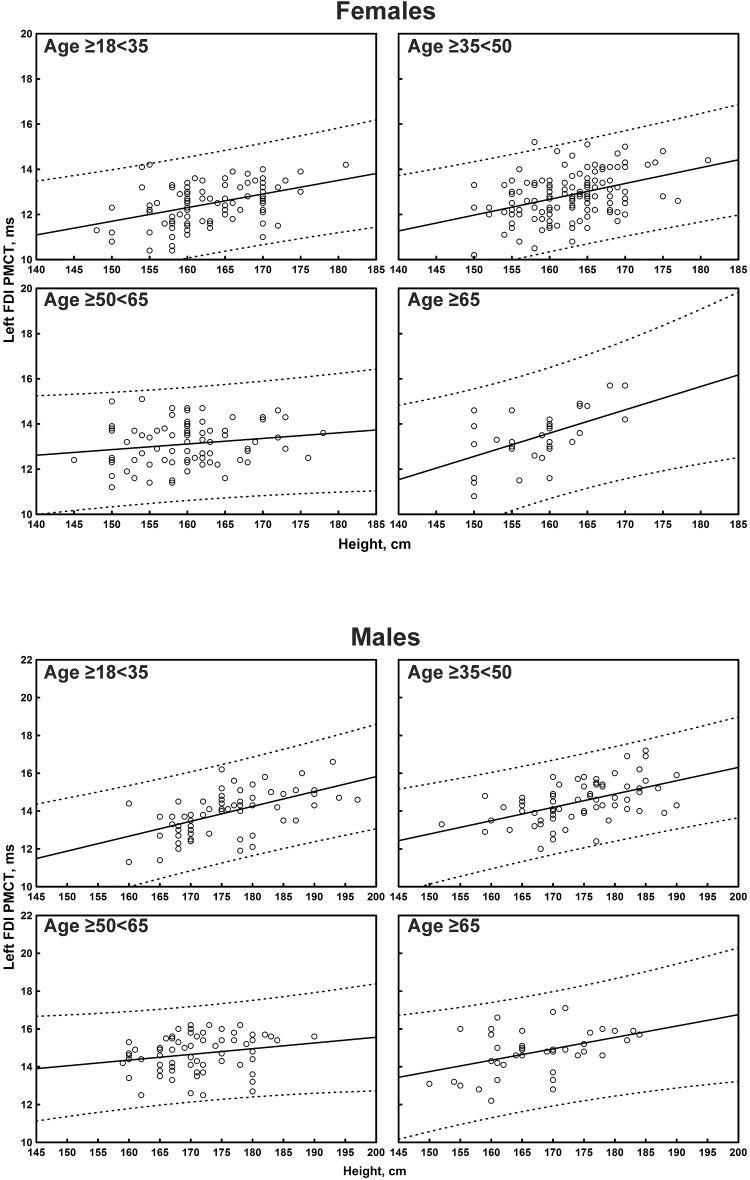
Correlation between age and left first dorsal interosseous muscle (FDI) peripheral motor conduction time (PMCT) in each age subgroup of participants, divided by sex. The continuous line is the regression line while the two dashed lines represent the limits of the area within which the 95% of points are expected.

**FIGURE 8:**
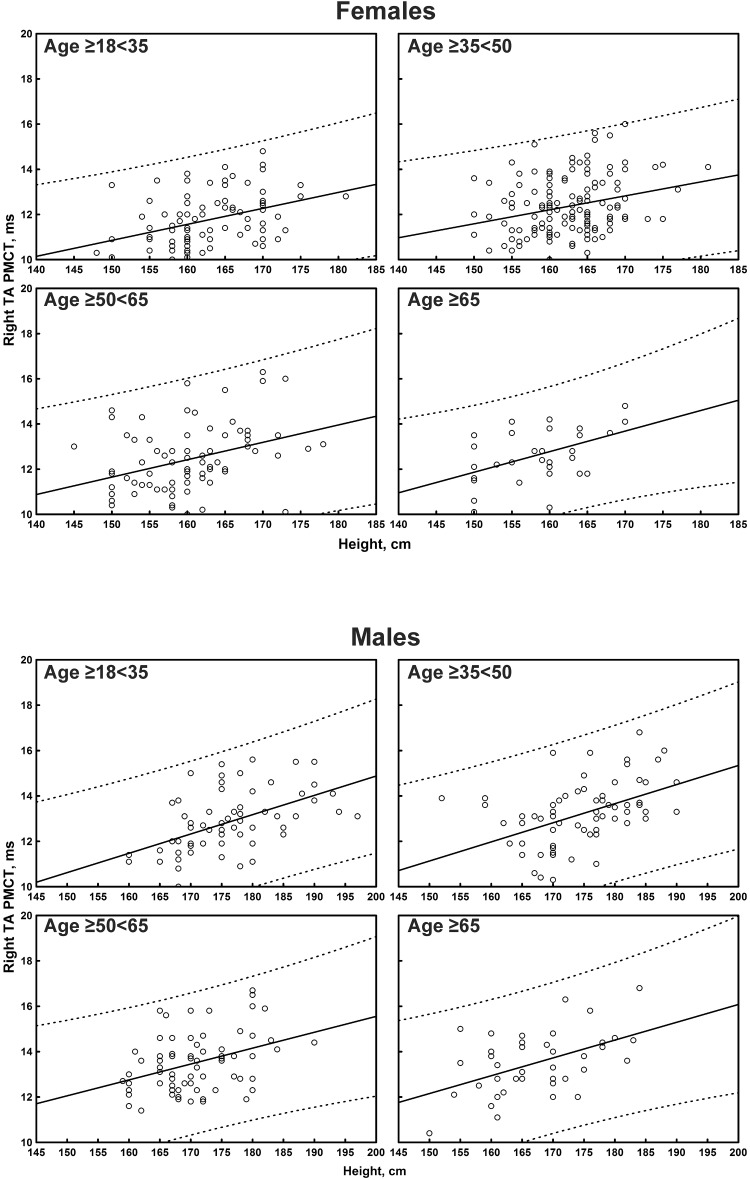
Correlation between age and right tibialis anterior muscle (TA) peripheral motor conduction time (PMCT) in each age subgroup of participants, divided by sex. The continuous line is the regression line while the two dashed lines represent the limits of the area within which the 95% of points are expected.

**FIGURE 9:**
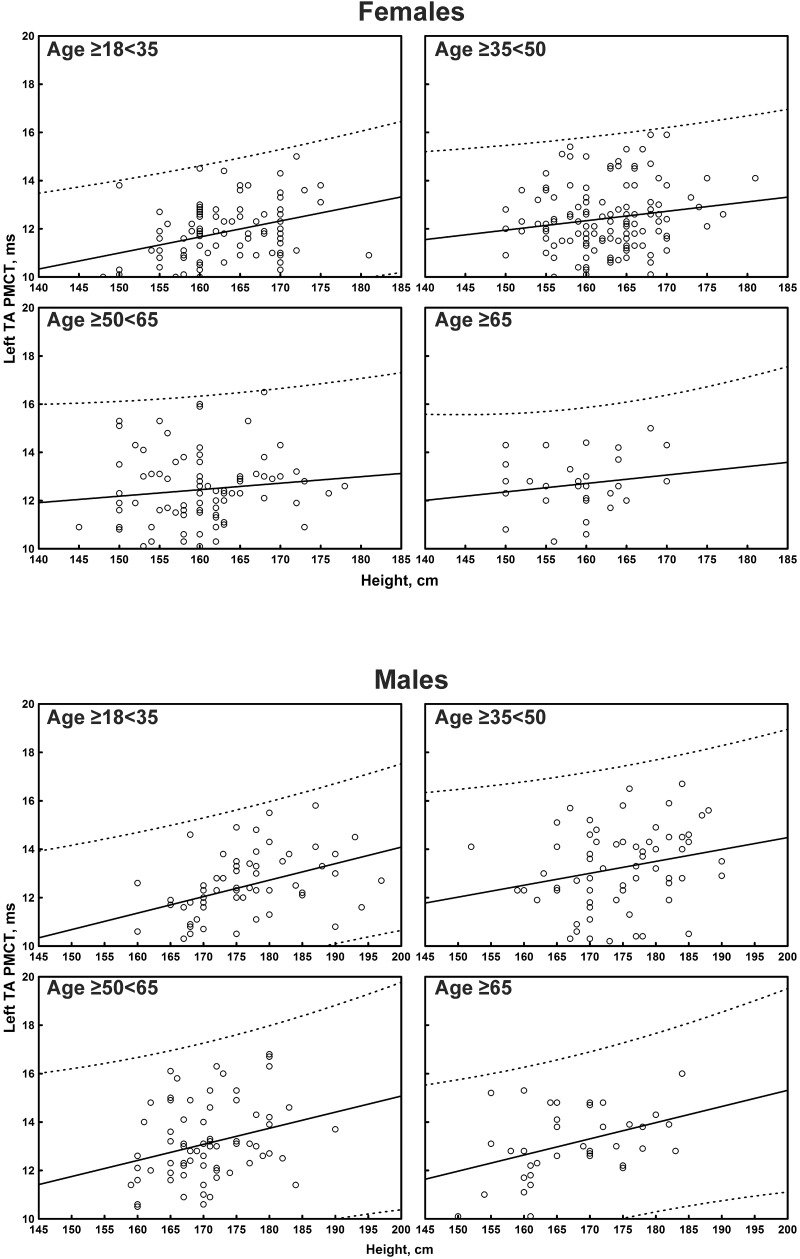
Correlation between age and left tibialis anterior muscle (TA) peripheral motor conduction time (PMCT) in each age subgroup of participants, divided by sex. The continuous line is the regression line while the two dashed lines represent the limits of the area within which the 95% of points are expected.

**FIGURE 10:**
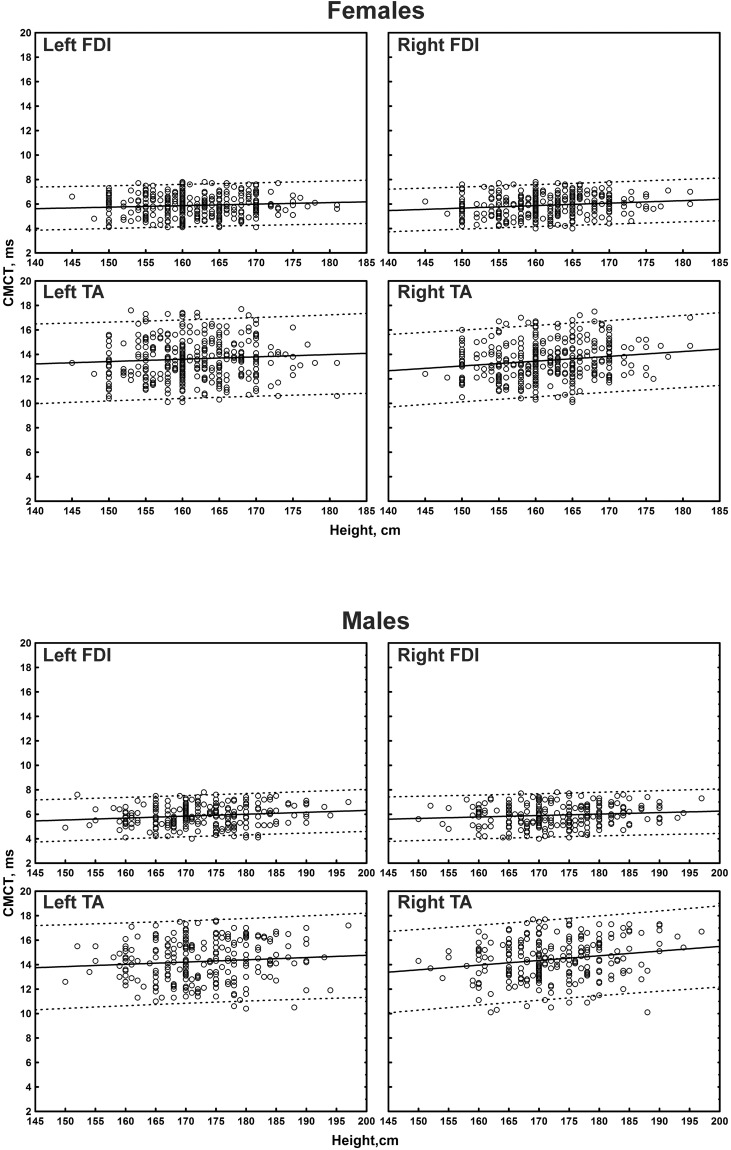
Correlation between height and right or left first dorsal interosseous muscle (FDI) and right or left tibialis anterior muscle (TA) central motor conduction time (CMCT) in participants, divided by sex. The continuous line is the regression line while the two dashed lines represent the limits of the area within which the 95% of points are expected.

The multiple linear regression analysis of the correlation between age, height, and sex, and all the TMS measures is shown in Table 4. Subgroup-specific graphs for MEP cortical latency and PMCT at the four limbs are shown in Figure 2–9, which illustrate their correlation with height, for each muscle in each age subgroup, further subdivided by sex. Figure 10 shows the correlation between height and CMCT from right or left FDI and TA in participants, divided by sex.

**Table 4:** General linear (multiple) regression analysis of the correlation between age, height, and sex and all the variables studied.

	Age		Height		Sex	
			
	partial correlation	*p* < ^∗^	partial correlation	*p* < ^∗^	partial correlation	*p* < ^∗^
**Right FDI**						
*MEP amplitude, mV*	-0.112		0.060		-0.069	
*MEP cortical latency, ms^∗^*	0.284	0.000001	**0.394**	0.000001	-0.212	0.000012
*PMCT, ms^∗^*	**0.383**	0.000001	**0.381**	0.000001	-0.281	0.000001
*CMCT, ms*	-0.075		0.099		0.044	
**Left FDI**						
*MEP amplitude, mV*	-0.129		0.073		-0.099	
*MEP cortical latency, ms^∗^*	**0.301**	0.000001	**0.415**	0.000001	-0.243	0.000001
*PMCT, ms^∗^*	**0.397**	0.000001	**0.405**	0.000001	**-0.324**	0.000001
*CMCT, ms*	-0.068		0.092		0.052	
**Right-Left difference**						
*MEP amplitude, mV*	0.015		-0.014		0.037	
*MEP cortical latency, ms*	-0.015		-0.018		0.041	
*PMCT, ms*	-0.004		-0.024		0.058	
*CMCT, ms*	-0.010		0.010		-0.008	
**Right TA**						
*MEP amplitude, mV*	-0.089		-0.086		-0.220	0.000004
*MEP cortical latency, ms^∗^*	**0.317**	0.000001	**0.433**	0.000001	-0.097	
*PMCT, ms^∗^*	**0.323**	0.000001	**0.404**	0.000001	-0.023	
*CMCT, ms*	0.119		0.206	0.000025	-0.094	
**Left TA**						
*MEP amplitude, mV*	-0.010		-0.038		-0.153	0.0095
*MEP cortical latency, ms^∗^*	0.231	0.0000008	0.265	0.000001	-0.062	
*PMCT, ms^∗^*	0.253	0.000001	0.274	0.000001	0.002	
*CMCT, ms*	0.075		0.101		-0.077	
**Right-Left difference**						
*MEP amplitude, mV*	-0.088		-0.055		-0.084	
*MEP cortical latency, ms*	0.063		0.159	0.00012	-0.026	
*PMCT, ms*	0.048		0.121		-0.025	
*CMCT, ms*	0.038		0.098		-0.011	


*Correlations with medium-to-large size (≥0.30) are indicated in bold lettering. ^∗^, Significant after bonferroni correction (non-significant *p*-values are not shown); FDI, first dorsal interosseous muscle; TA, tibialis anterior muscle; MEP, motor evoked potential; PMCT, peripheral motor conduction time; CMCT, central motor conduction time.*

A small non-significant correlation size was observed between MEP amplitude in the upper limbs and all the physical variables considered. In the lower limbs, a statistically significant correlation, but with a small-to-medium correlation size that did not resist to Bonferroni correction, was observed between MEPs amplitude and sex.

Motor evoked potential cortical latency at the four limbs correlated with age (medium-to-large correlation size for the left FDI and right TA; small-to-medium for the other limbs) and height (medium-to-large correlation size for the upper limbs and right TA, small-to-medium for the contralateral side). At the upper limbs, a significant correlation that passed the Bonferroni correction was observed between MEP cortical latency and sex (shorter in women), although with a small-to-medium correlation size. No correlation was evident between MEP cortical latency and sex at the lower limbs.

Peripheral motor conduction time at the four limbs positively correlated with age and height (medium-to-large correlation size for both FDI and right TA; small-to-medium for the contralateral muscle). At the upper limbs, PMCT correlated with sex, being shorter in women (medium-to-large correlation size for the left FDI; small-to-medium for the contralateral muscle), with a statistically significant difference, even after correction, bilaterally. A small non-significant correlation size was found with gender for the lower limbs.

Central motor conduction time correlated with both age and height when analyzed by a single regression. In particular, age negatively correlated (with small correlation coefficients) with CMCT from the upper limbs (right: *r* = -0.108, *p* = 0.009; left: *r* = -0.100, *p* = 0.015); height positively correlated (with small correlation coefficients) with CMCT from the upper limbs (right: *r* = 0.110, *p* = 0.008; left: *r* = 0.094, *p* = 0.024), while the correlation from the lower limbs appeared to be small-to-medium (right: *r* = 0.304, *p* < 0.001; left: *r* = 0.173, *p* < 0.001). However, when analyzed by multiple regression these significances disappeared, due to the correction for the multicollinearity within the dataset.

Regarding the difference between right and left side, a small non-significant correlation size was found for all TMS measures and the physical variables here considered at the four limbs. A statistically significant small-to-medium correlation size was evident for MEP cortical latency at the lower limbs, although it was not confirmed after Bonferroni correction.

## Discussion

### Main Findings

The main finding of this study is that individual features need to be considered for accurate MEP evaluation and meaningful interpretation. In particular, when reference values of MEP cortical latency and PMCT are used, the correlation with age, height and, to a lesser extent, sex must be taken into consideration. This approach will account for the unwanted variability associated with demographic and physical variables and allows for appropriate and reliable comparisons of MEPs, especially in studies with heterogeneous groups of participants. Accounting for the variability of MEP responses is imperative to demonstrate or confirm a clinical picture possibly due to a central nerve pathology and not to technical artifact, selection bias, or methodological error.

More in detail, we found a positive correlation of age and height with MEP cortical latency at the four limbs, supporting previous studies showing similar results (Booth et al., 1991). One of the underlying mechanisms is probably owing to the fact that age- and length-dependent changes affect the cervical and lumbo-sacral pools of spinal motoneurons differently (Tomlinson and Irving, 1977). Indeed, there is a progressive temporal dispersion of descending impulses with a less synchronized effect on the foot α-motoneurons (Rossini, 1988; Rossini and Caramia, 1988; Rossini et al., 1992). The cervical cord also receives much more cortico-spinal fibers per unit of muscle mass than the lumbo-sacral cord (Rossini, 1988; Rossini and Caramia, 1988; Rossini et al., 1992). Such physiological factors might thus influence the observed changes along the motor pathway. Moreover, MEP cortical latency have been shown to be different in males and females, with longer latencies in the former (Livingston et al., 2010). This result may be explained by the different average height between genders (Toleikis et al., 1991), thus explaining the differences of MEP cortical latency involving upper/lower limbs and males/females (Toleikis et al., 1991). However, in our study we found an additional independent effect of sex that might be based on other features, different from height (i.e., nerve diameter), although with our data we cannot speculate further on this point.

The present study also confirms those investigating the effect of aging and height on PMCT (Mayer, 1963; Kimura et al., 1975; Dorfman and Bosley, 1979; Matsumoto et al., 2012). Prior reports have demonstrated the importance of age-related and length-dependent peripheral nerve changes, such as progressive fiber loss and segmental demyelination (Lascelles and Thomas, 1966; Swallow, 1966; Rivner et al., 2001). In this context, it is worth to highlight that, unlike standing height (which decreases progressively with aging), knee height remains relatively stable during adulthood, making this measurement a good alternative for calculating stature, especially in older adults (Chumlea et al., 1985; Lera et al., 2005).

It is noteworthy that, although amplitude of the motor response is known to be subject to several physiological influences, we did not observe significant correlation of MEP size with any physical variable, except for a small-to-medium correlation with sex at the lower limbs. However, this finding was not observed for the upper limbs, likely reflecting the gender-specific regional fat distribution and its effects on electrophysiological recording. As in EMG studies, indeed, most gender differences in nerve conduction velocity are largely explained by height, whereas differences in amplitude can be due to body composition and fat distribution (Robinson et al., 1993; Buschbacher, 1998).

Regarding CMCT, it is known that in adults it does not significantly correlate with age (Claus, 1990; Eisen and Shtybel, 1990; Mano et al., 1992; Mills and Nithi, 1997). Conversely, based on the different length of the motor pathway, a relationship between CMCT and height can be expected. In particular, since the conduction distance from M1 to the cervical segment is shorter than the lumbar segment, many studies found that CMCT to the upper limb muscles had no or only a weak correlation with height, whereas CMCT to lumbar segments was correlated with height (Rossini et al., 1987, 2015; Chu, 1989; Claus, 1990; Ghezzi et al., 1991; Ravnborg and Dahl, 1991; Toleikis et al., 1991; Furby et al., 1992; Wochnik-Dyjas et al., 1997; Groppa et al., 2012; Udupa and Chen, 2013), without the influence from supraspinal sections (Claus, 1990). Formulae for calculating the upper limit of normal CMCT taking height into account have also been proposed (Claus, 1990).

Also in our study, CMCT appeared to be correlated with both age (negatively) and height (positively) when analyzed by a single regression; however, with a multiple regression analysis this significance disappeared, due to the correction for the multicollinearity within the dataset. The use of a multiple regression analysis, indeed, may probably explain the lack of a significant effect of height on conventional CMCT. Indeed, any height-related difference in intrathecal peripheral component is relatively small when compared with the differences in the more distal peripheral tract. However, even if our study suggests that height-related effects are small and non-statistically significant in these neurologically intact subjects, this might not be true in those with specific diseases, such as cauda equina disorders or severe peripheral neuropathy. In these cases, indeed, the evaluation of CMCT by means of paravertebral magnetic stimulation might not be sufficient to differentiate a cortico-spinal tract involvement from an intrathecal peripheral involvement. In pathological conditions, therefore, the effect of height becomes much more pronounced and an evaluation of CMCT with the F-wave method is mandatory. An alternative technique is the use of a modified coil, termed MATS (magnetic augmented translumbosacral stimulation), that activates the spinal roots at the conus medullaris level, thus making it possible to evaluate the CCCT for leg muscles (Matsumoto et al., 2010). Interestingly, using this coil in a sample of 51 Asian healthy volunteers, Matsumoto and coworkers showed that while there was a correlation between conventional CMCT and height, no correlation was present when the CCCT was considered (Matsumoto et al., 2010). However, unlike the present work, a multiple regression analysis was not performed (Matsumoto et al., 2010).

Overall, this matter remains still controversial, with some investigators showing that CMCT was independent of both height and age (Ugawa et al., 1989; Booth et al., 1991; Mano et al., 1992; Heald et al., 1993) and others demonstrating the opposite (Chu, 1989; Eisen and Shtybel, 1990; Furby et al., 1992) (for a recent comprehensive review, see Rossini et al., 2015). The reasons for such discrepant results remain unclear, although a reasonable explanation may be attributed to the different methods used across the studies and the demographic characteristics of the subjects. Notably, as mentioned, most of the previous reports adopted a simple regression analysis that, however, seems to be insufficient to analyze the effects of all physical variables on MEPs features. Conversely, a combined regression analysis provides a better prediction than each variable alone, as also demonstrated by studies using somatosensory (Allison et al., 1983; Chu, 1986) and visual evoked potentials (Celesia et al., 1987).

Finally, we did not find correlation between CMCT and sex or right-to-left difference (Claus, 1990; Toleikis et al., 1991; Furby et al., 1992; Mills and Nithi, 1997), in agreement with previously published reports (Eisen and Shtybel, 1990; Mills and Nithi, 1997), except for two. Chu (1989) compared two subgroups of female and male subjects with a homogenous height and found a gender difference in the leg CMCT, but not at the upper limbs. The other study reported a CMCT to the lower limbs marginally shorter in women than men, even controlling for differences in age and height (Tobimatsu et al., 1998).

### Clinical Implications

As a general rule, laboratory environment, technical set up, stimulation and recording protocols, and measurement procedures need to be all standardized to allow a proper comparison within and across subjects. For instance, TMS data are influenced by the intensity and the time course of the magnetic field, the pulse configuration, and the relative threshold of each volley to the direction of the induced current flow in the cortex. The shape of the stimulation coil is also important because it influences the spatial distribution of the magnetic field (Di Lazzaro et al., 2003, 2004).

For clinical examination, cut-off values that separate normal and abnormal measurements should be available in every laboratory, for each muscle and adjusted for age, height, and sex. The measurement should be judged as abnormal when a given value deviates 2 SD (or, more conservatively, 2.5) from the mean of the data obtained from the control group. A right-left comparison is also recommended, especially to detect subtle abnormality on one side. While often difficult, it is important to build up a set of control data that match the specific population to study, since sensitivity and specificity of measurements may be insufficient if this is not done.

### Strengths and Limitations

The recruitment of a large and homogenous sample, including elderly subjects, is the main strength of this study. Additionally, to the best of our knowledge, this is the largest “real-world” TMS study. As known, this type of studies allow the inclusion of a considerable number of subjects with a wide range of demographic features, thus realistically mimicking real-life practice settings (Zhang et al., 2019). Nonetheless, several limitations must also be acknowledged.

(i) Given that the study was conducted within a clinical environment, the sample could not be represented by healthy volunteers but by subjects (almost all out-patients) who, however, did not have any clinical and radiological evidence of a motor system disorder.
(ii) The sample of subjects was retrieved from a database containing all the TMS records collected in the Lab. Therefore, as in all retrospective studies, a selection bias cannot be entirely excluded, although the subjects were consecutive and carefully screened. In particular, the analysis of data was performed independently by two of the authors (MC and MP) and any discrepancy was discussed and resolved among all the authors to ensure consensus, as recommended (Makady et al., 2017).
(iii) The most precise estimation of the MEP size is through the amplitude ratio (the ratio between the maximal transcranially evoked MEP amplitude and the maximal distally evoked compound motor action potential). Moreover, to describe the stimulus-response characteristics, one should record MEPs over a wide range of intensity levels, both at rest and during contraction. However, even this if helpful in research to minimize the inter-trial and inter-subject variability, such a detailed assessment is not feasible in a routine clinical setting for diagnostic purposes (Groppa et al., 2012).
(iv) Central motor conduction time was not calculated by stimulating the peripheral nerve and eliciting the F-waves, but by magnetically stimulating the motor roots at their exit foramina (Mills and Murray, 1986) where the depolarizing threshold is the lowest (Rossini et al., 1987). This method overestimates the CMCT because the conduction time in proximal root segment between spinal cord and exit foramen is included. Moreover, with this method, spinal roots are not necessarily excited simultaneously (Claus, 1990). Nevertheless, unlike the F-wave technique, the method used here is applicable to most muscles (including TA) and is less painful (Chen et al., 2008). Moreover, as known, the electrical root stimulation only gives information on a relatively small sample of α-motoneurons and related motor axons (Groppa et al., 2012). Additionally, because conduction in the intraspinal part of the peripheral motor axons contribute to the central rather than the peripheral conduction time, the F-wave method can falsely increase CMCT in patients with nerve root lesions (Claus, 1990; Groppa et al., 2012). Finally, if F-wave persistence is low (normal for particular muscles, such as TA), the recorded F-wave sequence may not sample the fastest axons, thus producing a spuriously short CMCT (Rossini et al., 2015). Therefore, given that both approaches have pros and cons and that there is no optimal technique for all occasions (Rossini et al., 2015), many laboratories (including ours) prefer foraminal electromagnetic stimulation for routine diagnostic exams.
(v) An estimation of the peripheral nerve conduction velocity would have been useful to rule out a peripheral nervous system disease, although this goes beyond a routine TMS exam. Nevertheless, all subjects recruited did not have any sign or history of peripheral nerve pathology.
(vi) Limb length was not measured. This might result in some misinterpretations: for instance, MEP cortical latency can be prolonged in comparison to the contralateral limb as a result of pathological processes involving the cortico-motor pathway rather than explained by a subject’s longer limb. A previous study showed that when MEP cortical latencies were adjusted to an individual’s upper extremity length, no significant differences between limbs were observed (Livingston et al., 2010). Anyhow, we did not find correlations between side-to-side difference and any physical variable.
(vii) Finally, the timing of testing during the menstrual cycle and its potential effect on MEPs was not considered (Smith et al., 1999, 2002), although a conclusive remark on the relationship between TMS and hormonal status has not been firmly established.

## Conclusion

The relationship between TMS measures and individual features needs to be clearly defined. The ability of TMS to discriminate between a pathology affecting the motor system and a bias from external variables is mandatory in both clinical practice and research setting. In this scenario, an optimal interpretation of MEPs will be possible only by a comprehensive understanding of the relationship between the motor responses and these variables. Here, a considerable amount of TMS data over a more than a decade of daily clinical activity is provided.

Notwithstanding the mentioned limitations, in this large sample of subjects, age, height, and, sex were all important in defining and comparing MEPs. In particular, in order to construct MEPs normograms, age and body height had to be considered in the definition of the physiological range of MEP cortical latency and PMCT. Together with clinical, imaging, and other electrophysiological findings, CMCT can be considered as a reliable diagnostic and possibly prognostic translational marker of cortico-spinal conductivity in healthy subjects and in patients with neurological disorders.

## Data Availability

All relevant data are contained within the manuscript.

## Author Contributions

MC, GL, and MP conceived the study. RB, GP, and VDL designed and coordinated the study. GL, LV, and VP drafted the manuscript. RR, FF, and CV dealt with the clinical and neuroradiological assessment. MC, RR, and RF organized the database. RF performed the statistical analysis. LV, VP, FF, and RB reviewed the literature and wrote sections of the manuscript. CV, GP, VDL, and MP critically reviewed and finalized the manuscript. All authors contributed to manuscript revision, read, and approved the submitted version.

## Conflict of Interest Statement

The authors declare that the research was conducted in the absence of any commercial or financial relationships that could be construed as a potential conflict of interest.

## Abbreviations

CCCT: cortico-conus motor conduction time
CMCT: central motor conduction time
CNS: central nervous system
EMG: electromyography
F: female
FDI: first dorsal interosseous muscle
M: male
M1: primary motor cortex
MEPs: motor evoked potentials
n: number of subjects
PMCT: peripheral motor conduction time
SD: standard deviation
TA: tibialis anterior muscle
TMS: transcranial magnetic stimulation
